# The ATR inhibitor tuvusertib (M1774) sensitizes prostate carcinoma to natural killer cell-mediated cytotoxicity, which is further augmented by the IL-15 receptor superagonist N-803

**DOI:** 10.1007/s00262-025-04260-4

**Published:** 2025-12-19

**Authors:** Cailyn A. Lee, Jeffrey Schlom, James W. Hodge, Kellsye P. Fabian

**Affiliations:** Center for Immuno-Oncology, Center for Cancer Research, National Cancer Institute, National Institutes of Health, Bldg. 10, Rm 8B13, 9000 Rockville Pike, Bethesda, MD 20892 USA

**Keywords:** DNA damage response inhibition, ATR inhibition, Tuvusertib, Immunogenic modulation, Immune checkpoint blockade, IL-15, N-803, Prostate cancer

## Abstract

High levels of replication stress make cancer cells vulnerable to therapies targeting DNA damage response (DDR) proteins, such as ataxia telangiectasia and Rad3-related (ATR) protein kinase. ATR is a key mediator of the replication stress response and plays a vital role in maintaining genomic stability. This study investigates the immunomodulatory potential of the ATR inhibitor (ATRi) tuvusertib to improve the targeting of prostate cancer (PCa) by immunotherapy. Tuvusertib pretreatment sensitized human PCa cells (DU145 and 22Rv1) to PBMC-derived natural killer (NK)-mediated lysis. Flow cytometric analysis identified upregulation of death receptor tumor-necrosis factor-related apoptosis-inducing ligand receptor 2 (TRAIL-R2), NKG2D ligand ULBP-1, and programmed death-ligand 1 (PD-L1) as a potential immunomodulatory effect of tuvusertib. Enhanced avelumab-mediated antibody-dependent cell-mediated cytotoxicity and lysis by PD-L1 targeting high-affinity NK (PD-L1 t-haNK) cells were observed in tuvusertib-treated DU145. Tuvusertib sensitized cells to lysis by recombinant human TRAIL, and TRAIL signaling blockade reduced NK-mediated lysis of tuvusertib-exposed cells. RNA analysis revealed elevated p21 and reduced Bcl-xL transcript levels in tuvusertib-treated DU145, suggesting an accumulation of DNA damage and suppression of anti-apoptotic signaling, respectively. Pretreatment of NK cells with the interleukin-15 (IL-15) receptor superagonist N-803 (nogapendekin alfa inbakicept) further enhaced the lysis of tuvusertib-exposed cells. In vivo in the DU145 PCa xenograft model, tuvusertib and N-803 combination therapy demonstrated marked and significant antitumor efficacy relative to either monotherapy, eliciting superior tumor growth control and prolonging survival. Our findings support further investigation into the use of ATRi with immune checkpoint blockade and/or immune-stimulating agents in prostate cancer.

**Supplementary Information:**

The online version contains supplementary material available at 10.1007/s00262-025-04260-4.

## Introduction

Germline and somatic DNA damage response (DDR) gene mutations are harbored by approximately 12% and 23%, respectively, of patients with metastatic prostate cancer (PCa) [1], representing additional targetable elements for the treatment of PCa. The DDR protein ataxia telangiectasia and Rad3-related (ATR) protein kinase plays a key role in the cellular response to replication stress. Tumor cells’ reliance on ATR signaling due to high replication stress provides a rationale for ATR inhibition as a cancer therapy [2]. The potent and selective ATR inhibitor (ATRi) tuvusertib (M1774) has been shown to inhibit tumor cell proliferation and viability and is currently in clinical trials [3–6].

Immune checkpoint blockade (ICB) has had little success in PCa, a cold tumor characterized by low tumor mutational burden and T cell infiltration [7]. The downregulation of major histocompatibility complex class I (MHC-I) is one of the mechanisms underlying the poor response to ICB in PCa [8]. While MHC-I is important for the recognition of tumor cells by cytotoxic T lymphocytes [8], MHC-I^low/neg^ tumor cells are susceptible to lysis by natural killer (NK) cells, innate immune cells that lyse malignant cells by releasing lytic granules and engaging death receptors [9]. Additionally, NK cells can mediate antibody-dependent cell-mediated cytotoxicity (ADCC) through the interaction of CD16a and the Fc region of select tumor-targeted antibodies [10]. NK cell infiltration associates with improved outcomes in PCa [11]. Thus, therapies that improve NK cell infiltration or cytotoxic function, such as the interleukin-15 (IL-15) receptor superagonist N-803 [12, 13], could be an option for PCa.

Immunogenic modulation is the process by which select anticancer therapies phenotypically alter tumor cells, sensitizing them to immune-mediated killing and immunotherapy [14]. We examined tuvusertib’s ability to sensitize human PCa cells to immune cell killing and immunotherapy. Tuvusertib increased the susceptibility of DU145 and 22Rv1 to NK-mediated lysis in vitro. In the PD-L1^+^ DU145 cell line [15, 16], tuvusertib enhanced avelumab-mediated ADCC. Additionally, N-803 further augmented NK-mediated lysis of tuvusertib-treated DU145. The antitumor efficacy of the combination of tuvusertib and N-803 was demonstrated in the DU145 xenograft PCa model, with combination therapy exerting greater tumor growth control than either monotherapy. Altogether, our data provide a preclinical rationale for the use of tuvusertib with PD-L1-targeted therapies and/or immune-activating agents for PCa.

## Materials and methods

### Cell lines and materials

DU145 and 22Rv1 were obtained from American Type Culture Collection (Manassas, VA, USA), *Mycoplasma-*free, and used at low (< 22) passage number. Cells were cultured at 37 °C/5% CO_2_ in MEM and RPMI, respectively, supplemented with 10% fetal bovine serum, penicillin/streptomycin, gentamicin, sodium pyruvate, nonessential amino acids, and HEPES buffer. Tuvusertib (M1774) was provided through a Cooperative Research and Development Agreement (CRADA) between Merck KGaA (Darmstadt, Germany) and the National Cancer Institute (NCI; Bethesda, MD, USA). N-803 and programmed death-ligand 1 (PD-L1)‒targeting high-affinity NK cell line (t-haNK) were provided through a CRADA between ImmunityBio (Culver City, CA, USA) and the NCI.

### Tuvusertib treatment

Tuvusertib was solubilized in vehicle composed of 15% captisol (sulfobutylether-β-cyclodextrin; CyDex Pharmaceuticals, Lawrence, KS, USA) in 4.95 mM hydrochloric acid (HCl) and diluted in cell culture media. Unless otherwise specified, cells were treated with 0.5 µM tuvusertib for 48 h.

### Tumor lysis assay

Peripheral blood mononuclear cells (PBMCs) were obtained from healthy donors (HD) at the NIH Clinical Center Blood Bank (NCT00001846) and patients with PCa in a phase II clinical trial at the NCI (NCT00514072). NK cell isolation was performed using a human NK cell isolation kit (Miltenyi Biotec, Bergisch Gladbach, Germany), according to the manufacturer’s protocol. Prior to use in lysis assays, NK cells were cultured overnight at 37 °C/5% CO_2_ in RPMI supplemented as described above.

Tuvusertib-treated cells were co-incubated with HD-derived NK cells or PD-L1 t-haNK cells [17] at the indicated effector to target (E:T) ratios with or without 2 ng/ml avelumab (anti-PD-L1; EMD Serono, Boston, MA, USA) or 2.5 µg/ml anti‒tumor-necrosis factor-related apoptosis-inducing ligand (TRAIL) blocking antibody (clone RIK-2; Thermo Fisher, Waltham, MA, USA). Where specified, purified NK cells were exposed to 50 ng/ml N-803 overnight prior to co-incubation with target cells. For TRAIL-induced apoptosis, tuvusertib-treated cells were incubated with 20 ng/ml KillerTRAIL (Enzo Life Sciences, Farmingdale, NY, USA). All lysis assays were performed at 37 °C/5% CO_2_ in RPMI supplemented as described above. Cell lysis was measured using an impedance-based xCELLigence Real-Time Cell Analysis (RTCA) instrument (Agilent, Santa Clara, CA, USA). The cell index (CI) of each well was normalized to the CI at 1 h after the addition of the effector, and percent lysis was calculated using the following formula: percent lysis = [1 – (CI of sample well / average CI of control wells)] × 100. Control wells contained tumor cells that underwent the same treatment but were not exposed to an effector.

### Viability assays

Tumor and NK cells were exposed to 0, 0.001, 0.01, 0.1, 0.5, 1, or 10 µM tuvusertib in aforementioned culture conditions for the indicated durations. Cell viability was assessed via Acridine Orange/Propidium Iodide (AO/PI) staining (Logos Biosystems, Anyang, South Korea) or CyQUANT MTT assay (Thermo Fisher) following the manufacturer’s instructions. Data acquisition was performed using a LUNA-FX7 cell counter (Logos Biosystems) and a BioTek Synergy H1 microplate reader (Agilent), respectively.

### Flow cytometry

Live/dead fixable far red (Thermo Fisher) staining was used to exclude dead cells. Tuvusertib-treated cells were exposed to Human TruStain FcX Fc receptor blocking solution (BioLegend, San Diego, CA, USA) and stained with the following antibodies: TRAIL-R2-BV421 (clone B-K29) and PD-L1-PE (MIH1) from BD Biosciences (Franklin Lakes, NJ, USA) and ULBP-1-PE (170,818) from R&D Systems (Minneapolis, MN, USA).

To assess NK cell cytotoxic functions, HD-derived NK cells were isolated and rested overnight with or without 50 ng/mL N-803. DU145 were treated with 0.5 μM tuvusertib for 48 h and stained with 1 μM CellTrace Violet (Thermo Fisher) per 1 × 10^6^ cells for 20 min. The labelled DU145 cells were co-incubated with the NK cells at 0.5:1 E:T ratio for 4 h in the presence of GolgiStop and GolgiPlug (BD Biosciences) using the manufacturer’s recommended amounts. After which, the cells were stained with blue live/dead fixable dye (Thermo Fisher), exposed to Human TruStain FcX Fc receptor blocking solution (BioLegend), permeabilized using Foxp3 Transcription Factor Staining Buffer Set (Thermo Fisher), and stained with perforin-PE (clone δG9; BD Biosciences) and granzyme B-FITC (clone GB11; Biolegend). Cells were gated according to size, negative CellTrace Violet staining, and negative live/dead fixable dye to identify viable effector NK cells.

Data acquisition was performed using a BD LSRFortessa running FACSDiva software, and analyses were conducted using FlowJo V.10.9.0 (BD Biosciences).

### RNA analysis

Total RNA extraction from tuvusertib-treated cells was performed using the Qiagen RNeasy Mini Kit (Hilden, Germany). The Genomics Laboratory at Frederick National Laboratory for Cancer Research conducted the RNA analysis using the NanoString Technologies nCounter Tumor Signaling 360 Panel (Seattle, WA, USA). The nSolver Analysis software V4.0 (NanoString Technologies) and Ingenuity Pathway Analysis software V01-23–01 were used to conduct gene expression and pathway analyses.

### In vivo experiments

All animal studies were conducted in accordance with the NCI-Bethesda Animal Care and Use Committee‒approved animal protocol CIO-002 and utilizing ARRIVE reporting guidelines [18]. On day 0, 8–12 week old male NU/NU mice (Jackson Laboratory, Bar Harbor, ME, USA), housed in microisolator cages under pathogen-free conditions, were inoculated subcutaneously (s.c.) on the flank with a 5.0 × 10^6^ DU145 cells admixed with Matrigel 50% (v/v). Prior to treatment, animals with tumors that were < 80mm^3^ and > 190mm^3^ in size were excluded so that when animals were randomized into 4 groups (untreated control, N-803-, tuvusertib-, and combination-treatment, n = 7–8/group), the average tumor volume per group was 120–130 mm^3^. Starting on day 14, cyclical feeding of tumor-bearing mice with tuvusertib-containing chow (218.75 mg tuvusertib per kilogram of chow, which is equivalent to 35 mg/kg dose in a 25 g mouse that consumes 4 g of food a day; Research Diets, New Brunswick, NJ, USA) for 5 days followed by control chow for 2 days was performed. Mice were given weekly injections of N-803 (1 µg, s.c.) starting on day 14. Treatments were administered until the end of the study. Tumor volume was calculated using the formula (length x width^2^)/2. Treatments and measurements were performed unblinded. Mice were euthanized when an ethical limit, including tumor dimension reaching 20 mm, tumor volume exceeding 2000 mm^3^, tumor ulceration covering 50% of the surface, or weight loss exceeding 20%, was reached.

### Statistical analysis

Comparisons of two groups were performed via Student’s t-test. Comparisons of greater than two groups were performed via one-way or two-way analysis of variance with Tukey’s multiple comparisons test and Tukey’s post hoc analysis. The Mantel-Cox (log-rank) test was used to compare survival curves. P values below 0.05 were considered significant. Error bars depict standard error of the mean (SEM). Analyses were performed using GraphPad Prism V.10.4.0 (San Diego, CA, USA).

## Results

### Tuvusertib increases NK-mediated lysis of PCa cells

**Fig. 1:**
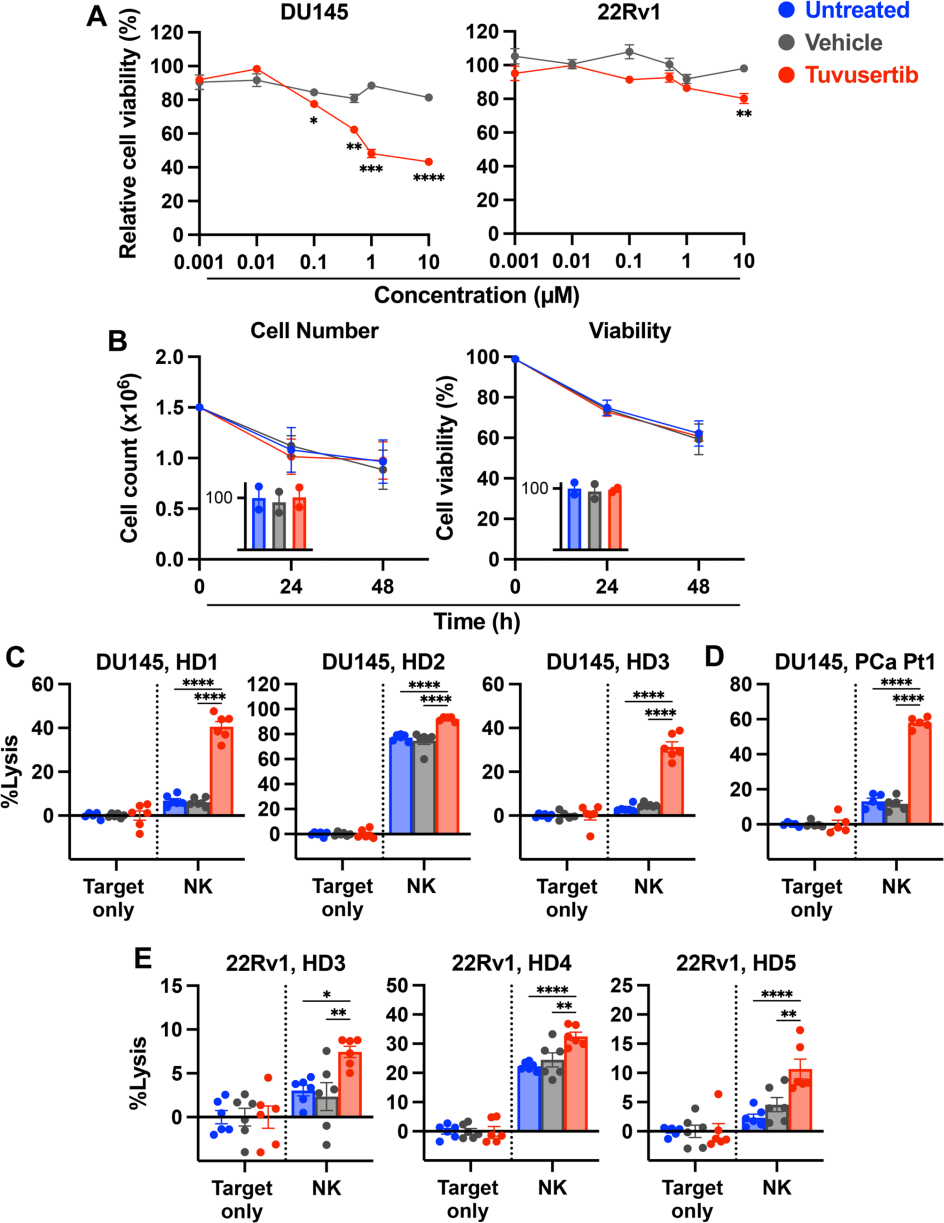
Tuvusertib increases NK-mediated lysis of PCa cells.** A** Human prostate carcinoma cell lines (DU145 and 22Rv1) were exposed to tuvusertib for 48 h at concentrations ranging from 1 nM to 10 µM. Cell viability relative to untreated cells was measured by MTT assay. **B** Cell count and viability of healthy donor NK cells (HD1, HD7) were measured by AO/PI staining following 24- or 48-h exposure to 0.5 µM tuvusertib. Insets report cell count and viability as a percent relative to untreated cells after 48-h drug exposure with each point representing a biological replicate. **C**–**E** DU145 and 22Rv1 cells were treated with 0.5 µM tuvusertib for 48 h prior to being washed and co-incubated with purified NK cells. Target only wells contain tumor cells without effector cells. Each point represents a technical replicate. Lysis of **C** DU145 cells by healthy donor (HD) NK cells (5:1 E:T ratio), **D** DU145 cells by prostate cancer patient (PCa Pt) NK cells (5:1 E:T ratio), and **E** 22Rv1 cells by HD NK cells (0.5:1 E:T ratio) at 24 h post-plating of effector cells. Statistical tests: A: Student’s t-test; B: one-way ANOVA with Tukey’s post hoc test; C–E: two-way ANOVA with Tukey’s post hoc test. Error bars, SEM. * p < 0.05, ** p < 0.01, *** p < 0.001, **** p < 0.0001. ANOVA, analysis of variance; AO/PI, acridine orange/propidium iodide; E:T, effector to target; MTT, 3-(4,5-dimethylthiazol-2-yl)-2,5-diphenyltetrazolium bromide

To evaluate tuvusertib’s activity against PCa, viability of DU145 and 22Rv1 cells was measured at different drug concentrations. Compared to captisol vehicle control, tuvusertib significantly reduced the relative viability of DU145 (p ≤ 0.018) and 22Rv1 (p = 0.001) at concentrations greater than or equal to 0.1 µM and 10 µM, respectively (Fig. 1A). The differential susceptibility to tuvusertib may be attributed to the disparate p53 status of the two cell lines, as others have shown that defects in *TP53* confer sensitivity to ATRi [19]. 22Rv1 harbors a wildtype *TP53* allele while DU145 possesses mutations in both *TP53* alleles [20]. The suboptimal drug concentration of 0.5 µM (185.16 ng/ml) was used to determine the capability of tuvusertib to exert immunogenic modification that will allow for improved immune cell targeting of the tumor cells without eradicating most of the tumor cells. This is within the range of the plasma concentrations observed in patients receiving the recommended dose for expansion in the first-in-human study (NCT04170153) [5] and the serum concentrations observed in mice receiving 218.75 mg tuvusertib per kilogram of chow (online supplemental Fig. S1).

To determine tuvusertib’s effect on NK cells, the number and viability of HD NK cells were measured following exposure to 0.5 µM tuvusertib for 24 or 48 h. Tuvusertib did not impact the number or viability of HD NK cells when compared to untreated control (Fig. 1B). Flow cytometric analysis revealed that tuvusertib exposure did not affect the phenotype of unstimulated or IL-2-stimulated NK cells (online supplemental Fig. S2).

To assess tuvusertib’s effect on the susceptibility of PCa cells to NK killing, tumor cells were treated with tuvusertib for 48 h, washed, and then co-cultured with NK cells purified from HD or PCa patient PBMCs. Unless otherwise noted, NK-mediated lysis of tuvusertib-treated cells was compared to lysis of vehicle-exposed cells. Given the inter-donor variability in PBMC-derived NK cells [21], lysis by NK cells from several donors was assessed. Evaluating NK-mediated lysis of tumor cells at several E:T ratios (online supplemental Fig. S3), demonstrated that 22RV1 and DU145 have different levels of baseline sensitivity to NK-mediated killing, which could in part be attributed to p53’s promotion of tumor cell recognition and killing by NK cells [22]. Hence, a 5:1 E:T ratio was utilized in the more recalcitrant DU145, while 0.5:1 was used in 22RV1. Tuvusertib significantly enhanced lysis of DU145 by HD NK cells (p < 0.0001; Fig. 1C). Patient-derived NK cells have been reported to be poorly cytotoxic in several cancer settings, including PCa [23, 24]. Nonetheless, co-culture of tuvusertib-treated DU145 with NK cells derived from a PCa patient showed increased lysis compared to vehicle-treated tumor cells (p < 0.0001; Fig. 1D). Tuvusertib also significantly increased lysis of another PCa cell line, 22Rv1, by HD NK cells (HD3 p = 0.0047, HD4 p = 0.0013, HD5 p = 0.0018; Fig. 1E). These data indicate that a sublethal dose of tuvusertib can sensitize PCa cells to NK-mediated killing.

In our previous lysis assays, tuvusertib was washed from the tumor cell culture prior to co-incubation with effector cells. To evaluate tuvusertib’s effect on NK cell cytolytic function, DU145 cells were co-incubated with HD NK cells in the presence of 0.5 µM tuvusertib. Tuvusertib did not have a detrimental effect on NK cell cytolytic capacity (online supplemental Fig. S4A).

### Tuvusertib upregulates the death receptor TRAIL-R2, immune checkpoint ligand PD-L1, and NKG2D ligand ULBP-1 in DU145 cells

**Fig. 2:**
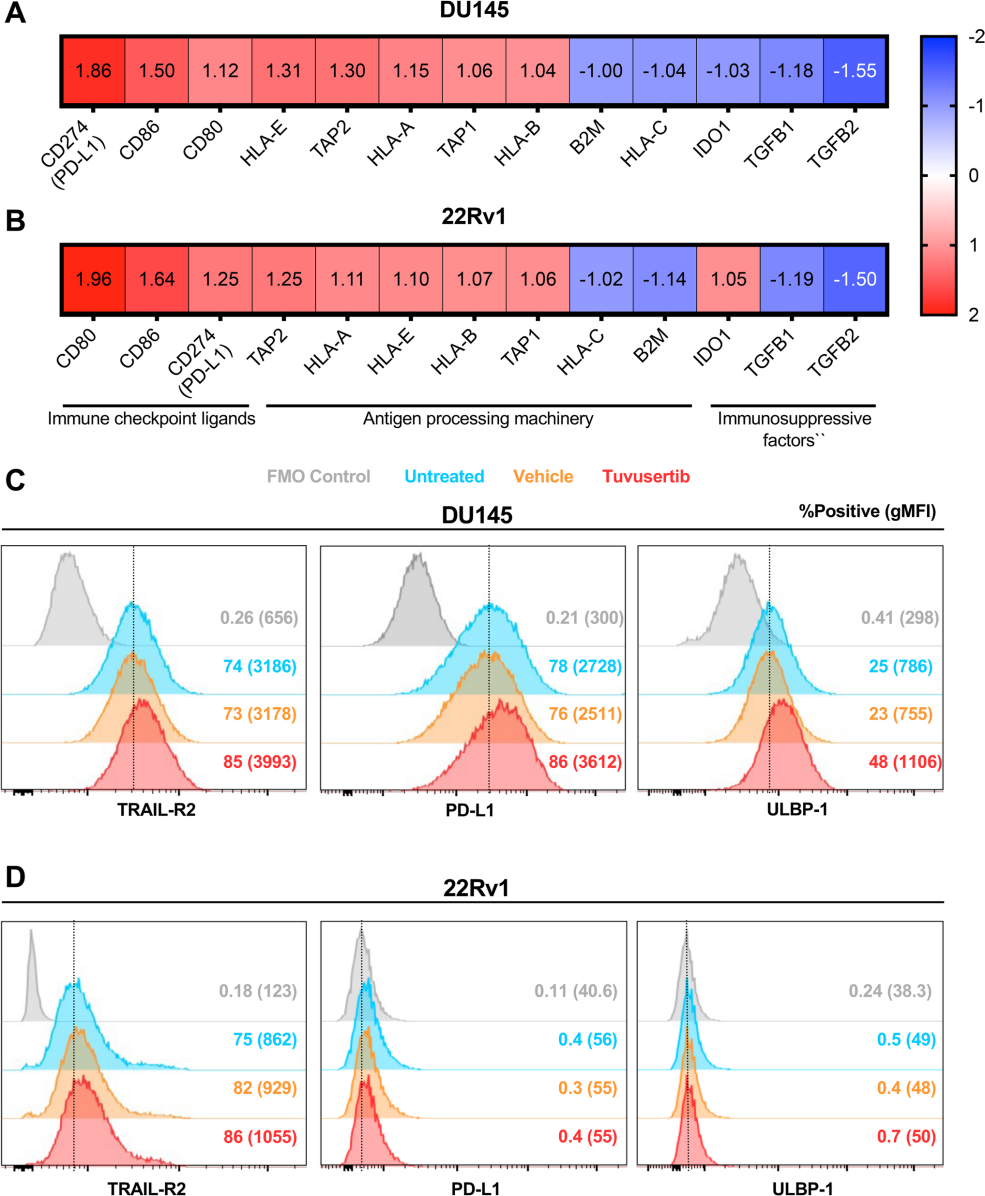
Tuvusertib immunomodulation upregulates the death receptor TRAIL-R2, immune checkpoint ligand PD-L1, and NKG2D ligand ULBP-1 in DU145 cells**.** Analysis of differentially expressed genes via NanoString Tumor Signaling 360 was performed on **A** DU145 and **B** 22Rv1 cells treated with tuvusertib as described in Fig. 1. Fold change relative to untreated cells is reported. Tuvusertib-treated **C** DU145 and **D** 22Rv1 cells were assessed for TRAIL-R2, PD-L1 (CD274), and ULBP-1 surface expression via flow cytometry. Percent of live cells expressing a given marker and the gMFI of live cells are reported. FMO, fluorescence minus one; gMFI, geometric mean fluorescence intensity; NKG2D, natural killer group 2 member D; PD-L1, programmed death-ligand 1;TRAIL-R2, tumor-necrosis factor-related apoptosis-inducing ligand receptor 2; ULBP, UL16-binding protein

Gene expression analysis of tuvusertib-exposed DU145 and 22RV1 cells showed a trend toward increased expression of immune checkpoint ligands, increased antigen processing machinery, and decreased expression of immunosuppressive factors (Fig. 2A,B). At the protein level, tuvusertib upregulated surface expression of the death receptor TRAIL-R2, immune checkpoint ligand PD-L1, and NKG2D ligand ULBP-1 in DU145 (Fig. 2C). TRAIL-R2 expression was also upregulated in tuvusertib-treated 22Rv1, but no change in expression of PD-L1 or ULBP-1 was observed in this cell line (Fig. 2D). TRAIL-R2 is of particular interest due to our previous report that identified TRAIL-R2 upregulation and TRAIL pathway engagement as mechanisms for the enhanced NK-mediated lysis of PCa cells exposed to another DDR inhibitor, the PARP inhibitor (PARPi) olaparib [16]. Meanwhile, TRAIL-R1 expression was not affected by tuvusertib treatment in both DU145 and 22RV1 cells (online supplemental Fig. S5).

### Tuvusertib sensitizes DU145 cells to PD-L1-targeted lysis by avelumab-mediated ADCC or PD-L1 chimeric antigen receptor (CAR)-directed killing with PD-L1 t-hANK cells

**Fig. 3:**
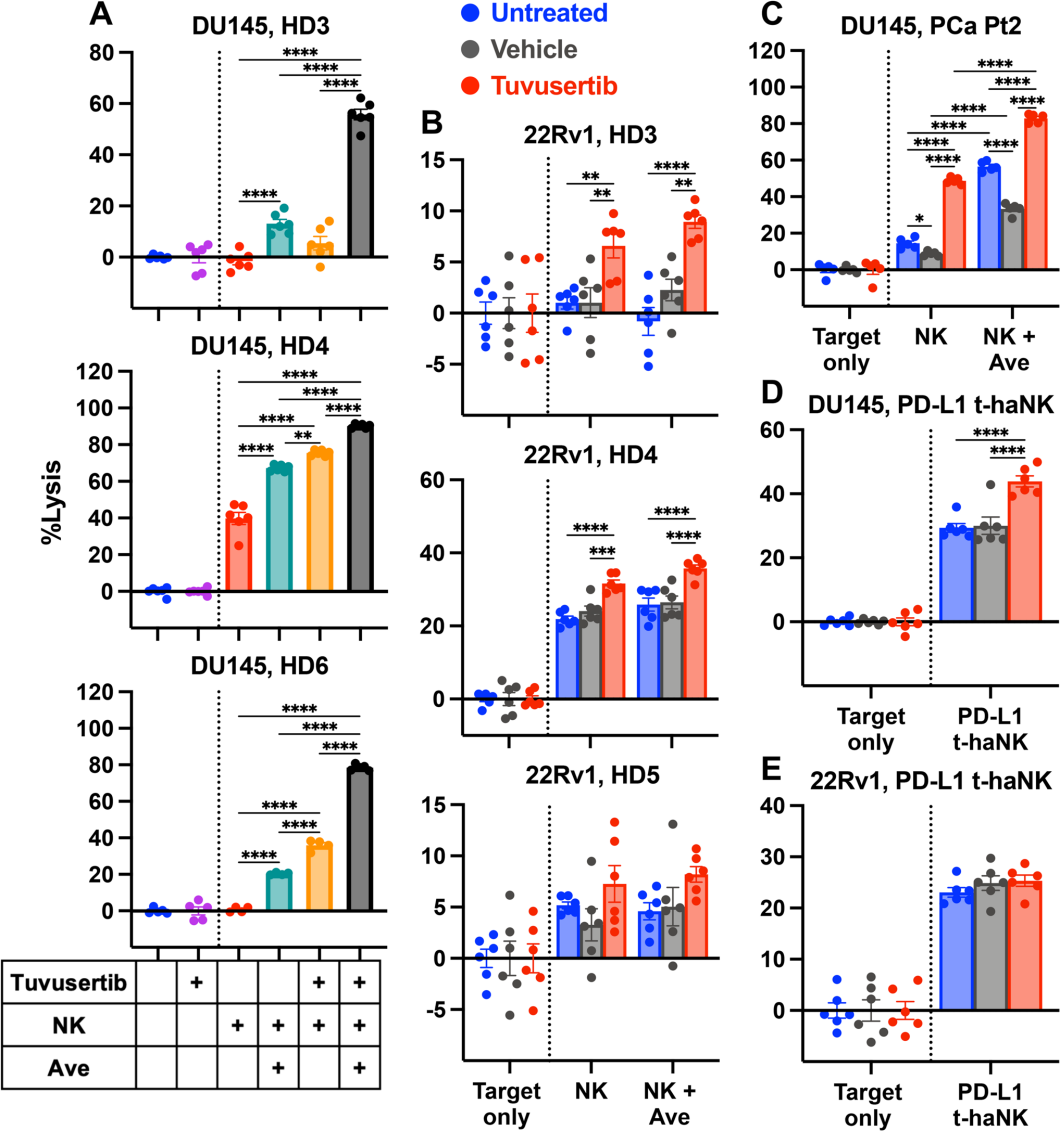
Tuvusertib sensitizes DU145 cells to PD-L1-targeted lysis by avelumab-mediated ADCC or PD-L1 CAR-directed killing with PD-L1 t-hANK. DU145 and 22Rv1 cells were treated with tuvusertib as described in Fig. 1, then washed prior to incubation with effector cells. Each point represents a technical replicate. **A**–**C** Avelumab-mediated ADCC was assessed in control and tuvusertib-treated DU145 and 22Rv1. Percent lysis of (**A**) DU145 cells by healthy donor NK cells (5:1 E:T ratio), **B** 22Rv1 cells by healthy donor NK cells (0.5:1 E:T ratio), and **C** DU145 cells by prostate cancer patient NK cells (5:1 E:T ratio) at 24 h post-plating of effector cells with or without avelumab is shown. **D**–**E** Percent lysis of control and tuvusertib-treated **D** DU145 and **E** 22Rv1 cells co-incubated with PD-L1 t-haNK cells (0.5:1 E:T ratio) at 24 h post-plating of effector cells. **D**–**E** The data shown are representative of two to three independent experiments. **B**–**E** Target only wells contain tumor cells without effector cells. Statistical tests: A: one-way ANOVA with Tukey’s post hoc test; B–E: two-way ANOVA with Tukey’s post hoc test. Error bars, SEM. * p < 0.05, ** p < 0.01, *** p < 0.001, **** p < 0.0001. ADCC, antibody-dependent cellular cytotoxicity; ANOVA, analysis of variance; Ave, avelumab; CAR, chimeric antigen receptor; E:T, effector to target; HD, healthy donor; NK, natural killer; PCa Pt, prostate cancer patient; PD-L1, programmed death-ligand 1; PD-L1 t-haNK, PD-L1 targeting high-affinity natural killer

Alongside their primary mechanisms of action, therapeutic antibodies of certain IgG isotypes can lyse tumor cells through ADCC [25]. We next evaluated whether the ADCC-mediating anti-PD-L1 IgG1 antibody avelumab [26] could further enhance the NK-mediated lysis of tuvusertib-treated DU145 and 22Rv1. NK killing (red; Fig. 3A) of untreated DU145 was significantly increased with the addition of avelumab (teal; p < 0.0001; Fig. 3A). Tuvusertib treatment of DU145 (orange) significantly enhanced NK-mediated lysis in two of three HD NK cells (HD4 and HD6; p < 0.0001; Fig. 3A). Tuvusertib and avelumab combination therapy (black) significantly enhanced NK-mediated lysis relative to tuvusertib alone and avelumab alone (p < 0.0001; Fig. 3A). Conversely, no increase in NK-mediated lysis of tuvusertib-treated 22Rv1 was observed with the addition of avelumab (HD3 p = 0.3872, HD4 p = 0.0754, HD5 p = 0.8789; Fig. 3B). Importantly, lysis of DU145 by PCa patient-derived NK cells was significantly increased by tuvusertib treatment (p < 0.0001) and the presence of avelumab (p < 0.0001), suggesting immunogenic modulation and avelumab-mediated ADCC, respectively (Fig. 3C). Tuvusertib and avelumab together further augmented NK-mediated lysis relative to either agent alone (p < 0.0001; Fig. 3C).

To further test tuvusertib’s effect on PD-L1-targeted therapy, we performed lysis assays with PD-L1 t-haNK, a PD-L1-directed CAR NK cell, as the effector cells (Fig. 3D, E). Tuvusertib pretreatment significantly increased lysis of DU145 by PD-L1 t-haNK 1.5-fold relative to vehicle (p < 0.0001; Fig. 3D). However, tuvusertib had no impact on the PD-L1 t-haNK-mediated lysis of the PD-L1^−^ 22Rv1 cell line (Fig. 3E). Although 22Rv1 cells lack PD-L1 expression, these data suggest that they nonetheless were targeted by PD-L1 t-haNK, likely through native NK receptor-mediated killing [17]. Taken together, these data suggest that tuvusertib increases the sensitivity of the PD-L1^+^ cell line DU145 to PD-L1-targeted therapies such as avelumab and PD-L1 t-haNK.

### Tuvusertib upregulates cyclin-dependent kinase inhibitor p21 and downregulates anti-apoptotic protein Bcl-xL in DU145 cells

**Fig. 4:**
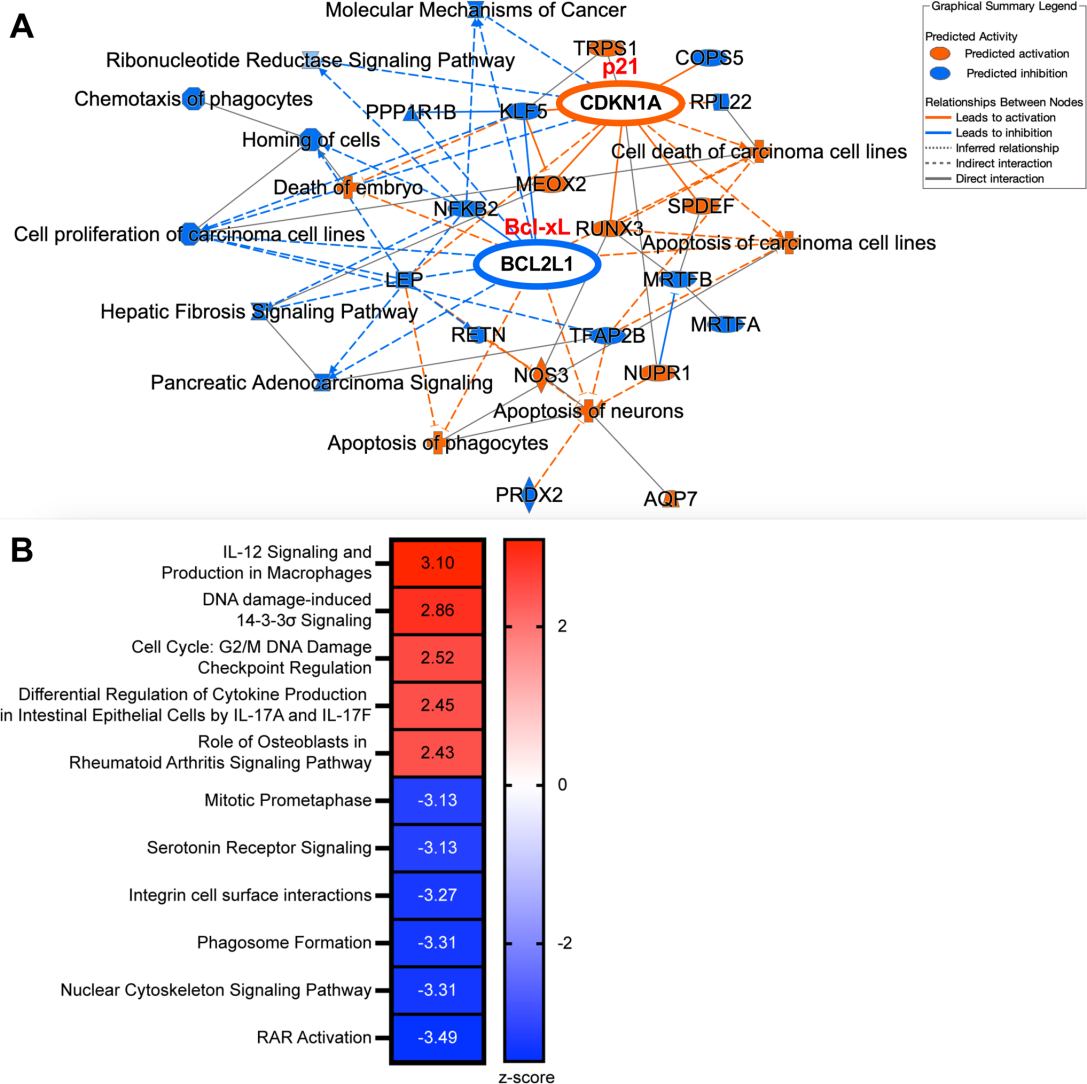
Tuvusertib upregulates cyclin-dependent kinase inhibitor p21 and downregulates anti-apoptotic protein Bcl-xL in DU145 cells. **A**–**B** Gene expression analysis via NanoString Tumor Signaling 360 was performed on DU145 cells treated with tuvusertib as described in Fig. 1. **A** Graphical summary of gene expression analysis and predicted biological activity, and **B** pathway enrichment analysis of tuvusertib-treated DU145 cells

Gene expression analysis of tuvusertib-treated DU145 revealed upregulation of *CDKN1A*, which encodes the cyclin-dependent kinase inhibitor p21, and downregulation of *BCL2L1*, which encodes the anti-apoptotic protein Bcl-xL; these molecules were predicted as nexuses of the modulated genes (Fig. 4A). p21 lies downstream of p53 in the ATM-CHK2-p53 pathway, and its canonical function is to trigger cell cycle arrest in G1 through inhibition of cyclin-dependent kinases such as CDK2 [27]. As a negative regulator of the intrinsic pathway of apoptosis, Bcl-xL prevents Bax and Bak activation and the associated permeabilization of the outer mitochondrial membrane [28]. Tuvusertib was also found to upregulate pathways related to the DNA damage response, such as DNA damage-induced 14–3-3σ signaling (Fig. 4B). Further validation of these data is required, especially the protein level of p21 and Bcl-xL. Nevertheless, the current data suggest that tuvusertib leads to an accumulation of DNA damage and renders cells more vulnerable to apoptosis.

### The TRAIL pathway plays a central role in the enhanced NK-mediated lysis of tuvusertib-exposed DU145 cells

**Fig. 5:**
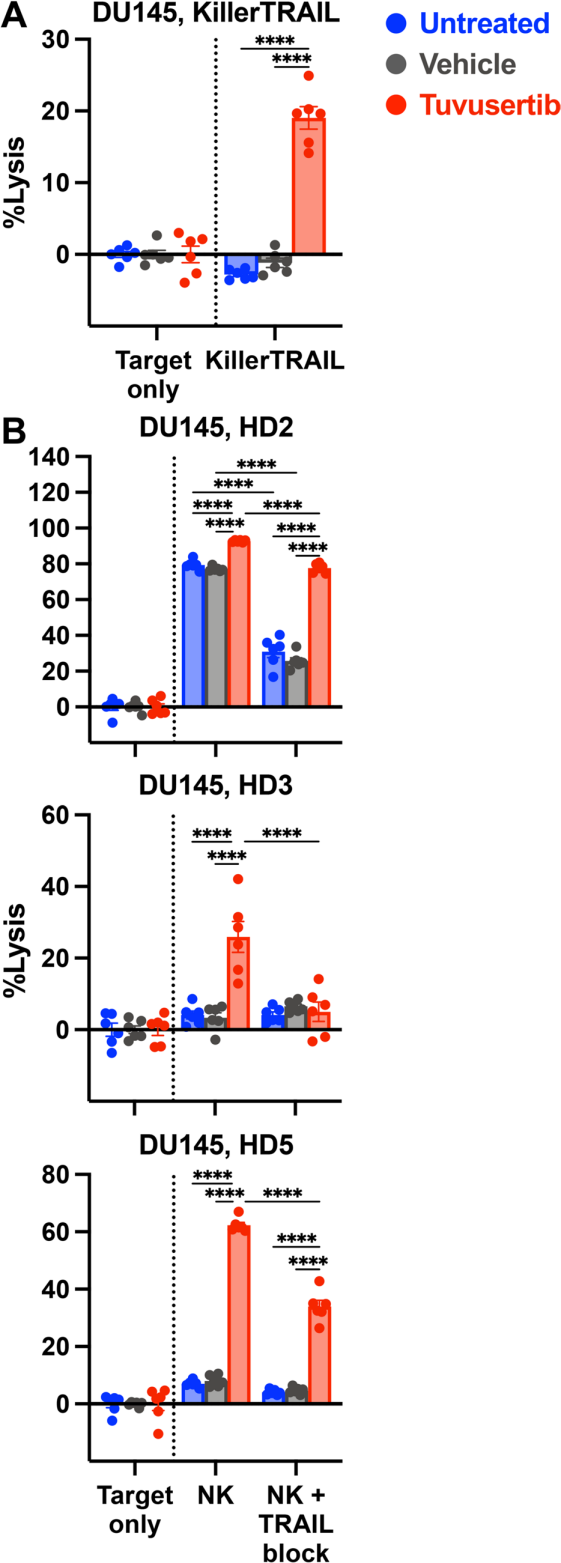
The TRAIL pathway plays a central role in the enhanced NK-mediated lysis of tuvusertib-exposed DU145 cells. DU145 cells were treated with tuvusertib as described in Fig. 1. Cells were washed prior to incubation with effector. Target only wells contained tumor cells without an effector. Each point represents a technical replicate. Lysis of tuvusertib-treated DU145 cells at 24 h after the addition of **A** KillerTRAIL or **B** NK cells (5:1 E:T ratio) derived from three different healthy donors in the presence or absence of anti-TRAIL blocking antibody. Statistical tests: two-way ANOVA with Tukey’s post hoc test. Error bars, SEM. **** p < 0.0001. ANOVA, analysis of variance; E:T, effector to target; HD, healthy donor; NK, natural killer; TRAIL, tumor necrosis factor-related apoptosis inducing ligand

Given the TRAIL-R2 upregulation in tuvusertib-treated DU145, we assessed the importance of TRAIL signaling in the augmented NK-mediated lysis of tuvusertib-treated DU145. Recombinant human TRAIL ligand, KillerTRAIL, induced the lysis of tuvusertib-treated DU145 (p < 0.0001; Fig. 5A). In contrast, TRAIL blockade significantly reduced NK-mediated lysis of tuvusertib-exposed DU145 (p < 0.0001; Fig. 5B). These data suggest that tuvusertib renders DU145 cells more vulnerable to TRAIL-mediated apoptosis and that TRAIL signaling plays a key role in the NK-mediated lysis of tuvusertib-treated DU145.

### IL-15 receptor superagonist N-803 further enhances the NK-mediated lysis of tuvusertib-treated DU145

**Fig. 6:**
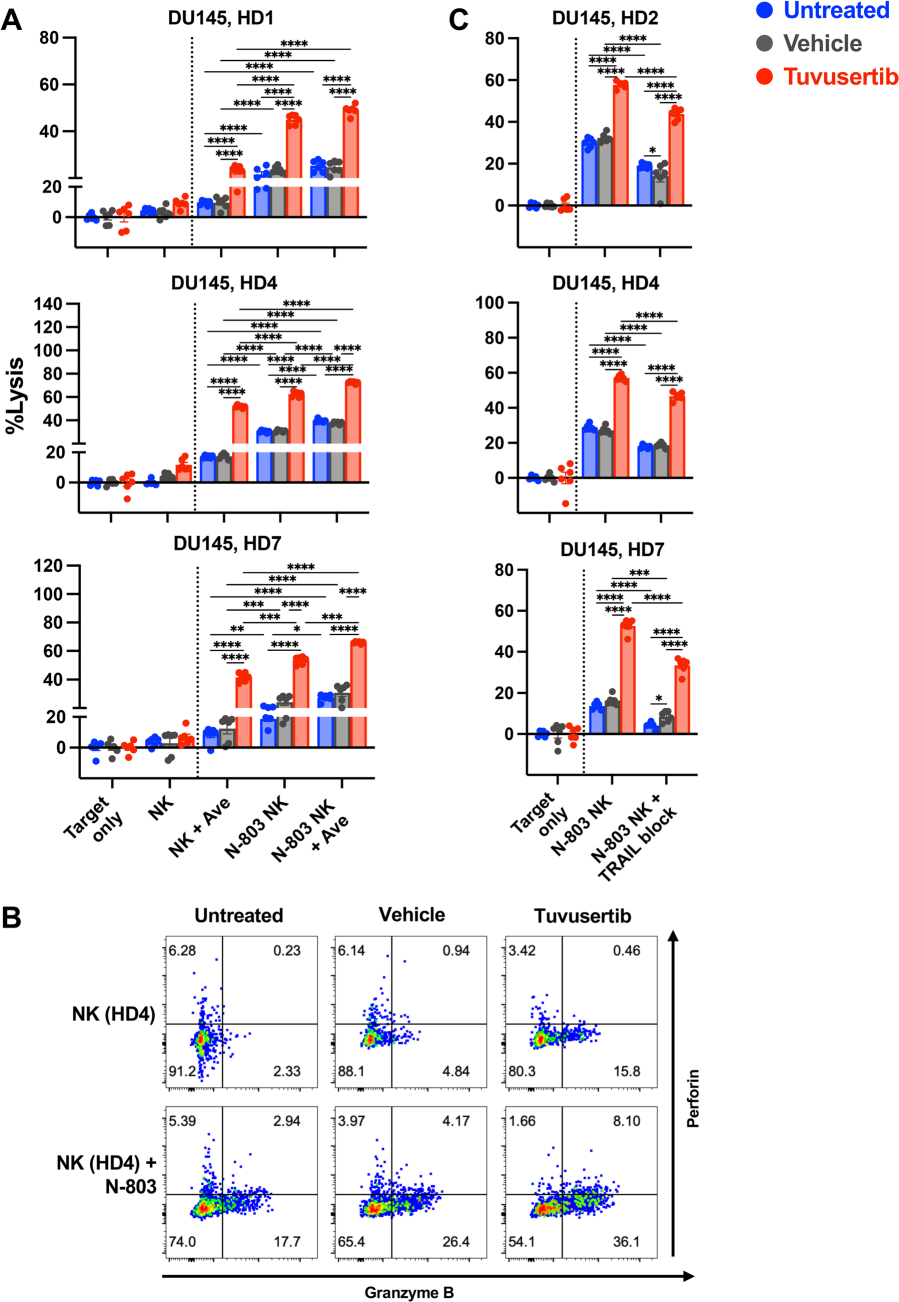
IL-15 receptor superagonist N-803 further enhances the NK-mediated lysis of tuvusertib-treated DU145. **A** Tumor cells were treated with tuvusertib as described in Fig. 1 prior to being washed and co-incubated with NK cells. NK cells were pretreated with 50 ng/ml N-803 overnight prior to use. Target only wells contained tumor cells without NK effector cells. Each point represents a technical replicate. N-803-enhanced NK-mediated lysis of DU145 cells (0.5:1 E:T ratio) in the presence of avelumab (anti-PD-L1). **B** Tumor cells and NK cells were prepared as described in A, with the tumor cells labeled with CellTrace Violet prior to co-incubation with the NK cells (0.5:1 E:T ratio) for 4 h. NK cells were assessed for intracellular expression of perforin and granzyme B via flow cytometry. **C** N-803-enhanced NK-mediated lysis of DU145 cells (0.5:1 E:T ratio) is partially blocked by anti-TRAIL monoclonal antibody. Lysis at 24 h following the addition of effector cells is shown. Statistical tests: two-way ANOVA with Tukey’s post hoc test. Error bars, SEM. * p < 0.05, ** p < 0.01, *** p < 0.001, **** p < 0.0001. ANOVA, analysis of variance; Ave, avelumab; E:T, effector to target; HD, healthy donor; NK, natural killer; PD-L1, programmed death-ligand 1﻿; TRAIL, tumor necrosis factor-related apoptosis inducing ligand

The ability of the IL-15 receptor superagonist N-803 to heighten NK cell cytolytic activity has been previously demonstrated [12, 13]. We observed similar enhancements in NK-mediated lysis of DU145 with N-803 pretreatment of NK cells (significance bar not shown; Fig. 6A). Nonetheless, tuvusertib further augmented N-803-enhanced NK-mediated lysis of DU145 (p < 0.0001; Fig. 6A). In addition, of the target cells that were still intact after a 24-h co-incubation with N-803-enhanced NK cells, a higher percentage of tuvusertib-treated DU145 cells were in the early apoptotic stage when compared to the untreated and vehicle-treated targets (online supplemental Fig. S6). The improved lysis of tuvusertib-exposed DU145 was associated with the increased cytotoxic function of the N-803-enhanced NK cells. Specifically, in co-cultures of NK cells with tuvusertib-treated DU145, the frequency of N-803-stimulated NK cells that express granzyme B and co-express granzyme B with perforin increased by up to three fold when compared to their counterparts that were incubated with control target cells (Fig. 6B). In the co-cultures, TRAIL expression by NK cells was increased by N-803 but was not influenced by what pre-treatment the target DU145 cells received (online supplemental Fig. S7).

Further increases in N-803-enhanced NK-mediated lysis of tuvusertib-exposed DU145 were observed with the addition of avelumab-mediated ADCC in assays using NK cells from two of three HDs (HD1 p = 0.2271, HD4 p < 0.0001, HD7 p = 0.0003; Fig. 6A). Lastly, TRAIL blockade significantly reduced the N-803-enhanced NK-mediated lysis of tuvusertib-exposed DU145 (p < 0.0001), suggesting that the TRAIL pathway occupies a central role in N-803-enhanced NK-mediated lysis of tuvusertib-treated cells (Fig. 6C). These data suggest that N-803 could be combined with tuvusertib with or without avelumab to maximize NK killing of PCa cells.

Lastly, assays in which DU145 cells were co-incubated with N-803-treated NK cells in the presence of tuvusertib demonstrated that tuvusertib does not negatively affect the cytolytic function of N-803-treated NK cells (online supplemental Fig. S4B).

### Combination therapy with tuvusertib and N-803 elicits an anti-tumor effect in the DU145 PCa xenograft model

**Fig. 7:**
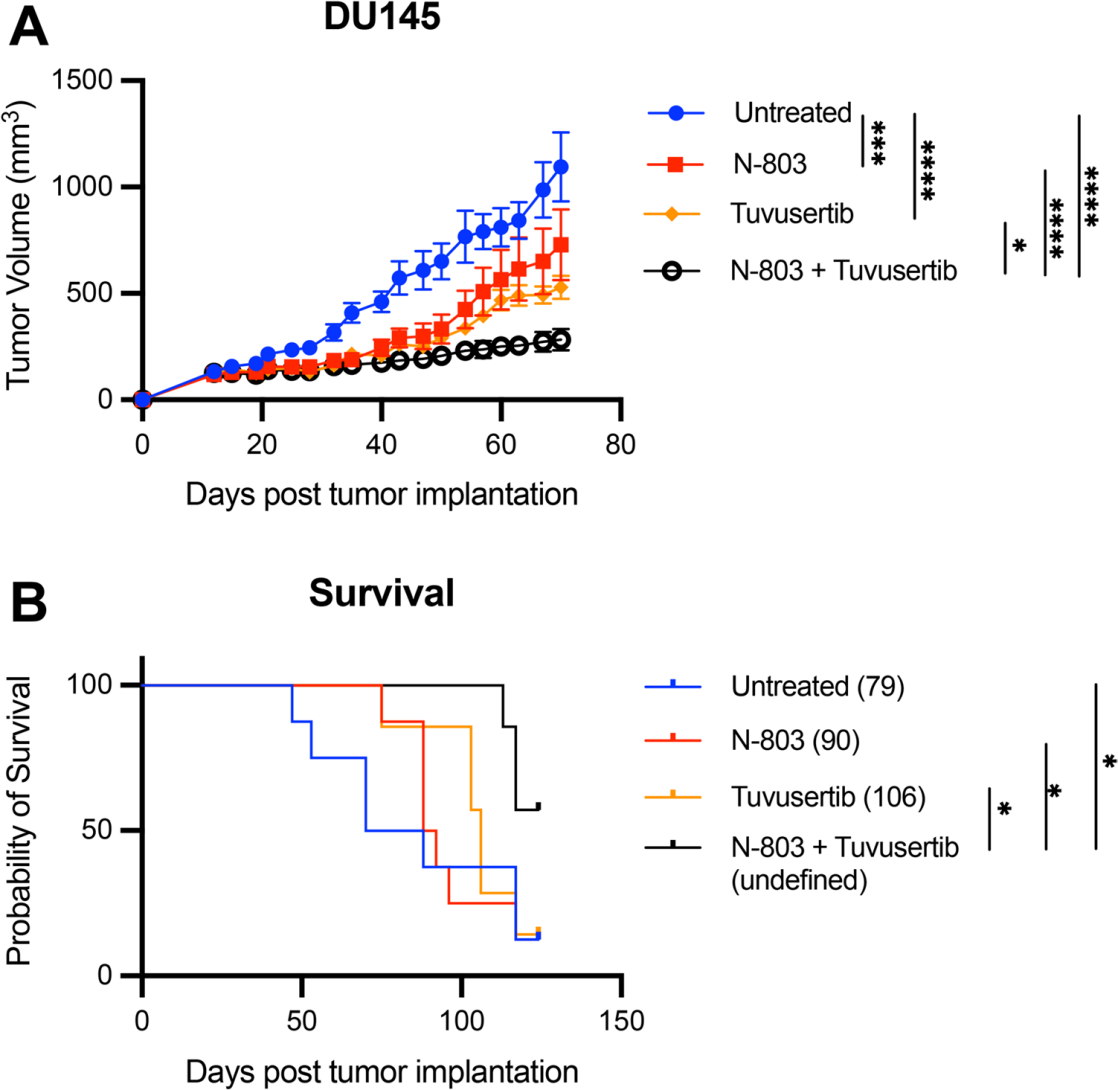
Combination therapy with tuvusertib and N-803 elicits anti-tumor effect in the DU145 PCa xenograft model. Male NU/NU mice (8–12 weeks old; n = 7–8/group) were implanted with 5 × 10^6^ DU145 cells on the right flank on day 0. Since the combination tested was novel, a small sample size was utilized. On day 14, tuvusertib was introduced in the animal feed (218.75 mg/kg) at 5 days on/2 days off cycles while N-803 (1 µg) was administered weekly by subcutaneous injections. A total of 16 treatment cycles were provided and the experiment was terminated when the majority of the mice reached ethical limits on day 125. Tumor growth was monitored. **A** Mean growth curves. **B** Animal survival was followed, with numbers in parentheses indicating the median overall survival. Statistical tests: Tumor growth: Two-way ANOVA with Tukey’s post hoc test; Survival: Mantel-Cox test. Error bars, SEM. * p < 0.05, ** p < 0.01, *** p < 0.001, **** p < 0.0001. ANOVA, analysis of variance

To evaluate the efficacy of the N-803 and tuvusertib combination in vivo, a xenograft model of DU145 PCa was utilized. DU145 cells were implanted s.c. in the flank of athymic nude mice, which lack T and B cells but retain normal levels of NK cells. Endogenous murine NK cells from NU/NU mice are capable of mediating antitumor activity against human cancer cell lines [29] and can be promoted by N-803 [13]. Starting on day 14 post-tumor inoculation, tuvusertib was orally administered through the food at 218.75 mg tuvusertib per kilogram of food (equivalent to 35 mg/kg body weight dose) for a cycle of 5 days, followed by 2 days with a control diet. N-803 was given as 1ug s.c. injections weekly. This treatment cycle was continued until the end of study. Monotherapy with tuvusertib or with N-803 significantly improved tumor growth control when compared with untreated animals (p = 0.0002 and p < 0.0001, respectively; Fig. 7A). Tuvusertib and N-803 combination treatment further enhanced anti-tumor efficacy and significantly inhibited tumor growth when compared with no treatment (p < 0.0001), N-803 monotherapy (p < 0.0001), and tuvusertib monotherapy (p = 0.0230; Fig. 7A). Furthermore, only the combination treatment with tuvusertib plus N-803 significantly prolonged the survival of the DU145-bearing mice, with more than half of the animals still on study after 125 days post-tumor implantation (Fig. 7B). Overall, these studies demonstrate that the combination of immunogenic-modulating tuvusertib with N-803 can synergize to promote improved anti-tumor effects in vivo.

## Discussion

Tuvusertib is an ATRi that has been shown to induce DNA damage and suppress tumor cell proliferation and viability [3, 4]. Preclinical studies involving tuvusertib have been focused on the ATRi’s direct cytotoxicity and have demonstrated its synergy with other DNA-targeted agents and MEK1/2 inhibitors [4, 30] We report for the first time that a sublethal dose of tuvusertib can act as an immunogenic modulator that reprograms PCa cells to become more susceptible to subsequent immunotherapy.

Accumulating immunological data suggest that prostatic NK cells may be more important than T cells in PCa [11, 31]. NK cells have been associated with improved patient outcome [11] and improved distant metastasis-free survival [31]. Hence, strategies to harness NK activity may be an essential component in treating PCa. Here we showed that tuvusertib improved the targeting of tumor cells by PCa patient-derived NK cells (Figs. 1D and 3C). Our data and previous work in NK cells from chordoma patients [12] contrast with earlier reports that NK cells from cancer patients have poor cytotoxicity [23, 24].

In addition to exacerbating replication stress, ATRis have also been reported to trigger endoplasmic reticulum (ER) stress [32]. We hypothesize that the phenotypic changes observed after tumor exposure to tuvusertib are partially mediated by the induction of the endoplasmic reticulum stress response pathway. ER stress induction in tumor cells can lead to the upregulation and translocation of proteins that promotes immune recognition and targeting of the tumor cells [33], including ligands for the NK cell-activating receptor NKG2D [34] and TRAIL receptors [35]. Here, we demonstrated that tuvusertib treatment increased tumor cell lysis by HD and PCa patient NK cells and is associated with increased surface expression of ULBP-1, an NKG2D ligand [36], on DU145 cells and the death receptor TRAIL-R2 [10] on both DU145 and 22Rv1 cells (Fig. 2C,D). The importance of TRAIL signaling in the NK-mediated lysis of ATRi-treated cells was supported by data showing that tuvusertib increased sensitivity to TRAIL-mediated apoptosis and TRAIL signaling blockade decreased NK-mediated lysis of tuvusertib-treated DU145 (Fig. 5). Since TRAIL-R1 expression was not affected by tuvusertib treatment (online supplemental Fig. S5), TRAIL-R2 may be the main receptor mediating the TRAIL pathway-related effect of tuvusertib. Further, tuvusertib decreased Bcl-xL transcript levels in DU145 cells (Fig. 4A). Greater Bcl-xL expression has been associated with resistance to TRAIL-induced apoptosis in breast and pancreatic cancer cells [37, 38], and inhibition or knockdown of Bcl-xL has been demonstrated to sensitize select TRAIL-resistant pancreatic cancer cell lines to TRAIL-mediated apoptosis [38, 39]. Additionally, higher expression of anti-apoptotic protein Bcl-xL has been associated with higher Gleason-grade PCa [40], suggesting its merit as a therapeutic target.

There is no consensus on ATRi’s effect on PD-L1 expression on malignant cells. While some studies showed reduced PD-L1 protein expression with ATRi [41], others have found the opposite. One study showed that ATRi with small molecule inhibitors or ATR-targeted siRNA augmented expression of PD-L1 in murine and human small cell lung cancer (SCLC) cell lines via the cGAS/STING pathway [42]. In another study, tumor biopsies from patients with ovarian and endometrial cancer receiving the ATRi elimusertib showed post-treatment increases in PD-L1 expression, albeit not statistically significant [43]. We observed PD-L1 upregulation in tuvusertib-treated DU145 (Fig. 2C), which subsequently allowed for improved targeting by PD-L1-directed therapies, such as avelumab (Fig. 3A,C) and PD-L1 t-haNK (Fig. 3D). Our data are in agreement with previous works that demonstrated improved antitumor efficacy with ATRi and anti-PD-L1 combination therapy. In some murine cancer models, the combination therapy resulted in enhanced tumor growth control [41, 42], associated with increased total and CD8^+^ cytotoxic T-cell infiltration in the tumor [42]. ATRi has also been shown to enhance the antitumor efficacy of radiotherapy and anti-PD-L1 combination therapy in murine hepatocellular carcinoma and colorectal cancer models [44, 45].

More broadly, small-molecule inhibitors of PARP, WEE1, and HDAC have been shown to sensitize tumor cells to avelumab-mediated ADCC [16, 46, 47]. Hence, we explored the potential of combining tuvusertib with avelumab and found that combination therapy increased NK-mediated lysis of PCa cells relative to either monotherapy, suggesting that avelumab-mediated ADCC can be harnessed to maximize NK-mediated lysis of ATRi-treated cells (Fig. 3A,C). However, previous data suggest that there may be variability in responses to concurrent tuvusertib and avelumab treatment based on the patient’s CD16a genotype. CD16a receptors with valine (V) rather than phenylalanine (F) at position 158 have greater affinity for IgG [48] and NK cells from individuals with the V/V genotype were found to mediate superior ADCC in vitro [49, 50]. Additionally, patients with the V/V genotype are more likely to respond to rituximab [51].

N-803 is an IL-15 receptor superagonist that is FDA approved for use in combination with Bacillus Calmette-Guérin (BCG) for BCG-unresponsive non-muscle invasive bladder cancer (NMIBC) with carcinoma in situ with or without papillary tumors [52]. Multiple studies have demonstrated the ability of N-803 to enhance NK cell cytolytic activity against several cell lines, including DU145 [13, 16]. We observed that N-803 pretreatment of NK cells further augmented NK-mediated lysis of tuvusertib-treated DU145 (Fig. 6A). The enhanced NK-mediated tumor lysis promoted by tuvusertib was not only due to immunogenic modulation on tumor cells but was also associated with increased perforin and granzyme B expression on the N-803-stimulated NK cell population when co-cultured with tuvusertib-treated cells (Fig. 6B). The synergy between tuvusertib and N-803 was confirmed in vivo in DU145 tumor-bearing mice in which the combination therapy outperformed either monotherapy at suppressing tumor growth and prolonging survival (Fig. 7). Furthermore, the triple combination of tuvusertib, N-803, and avelumab yielded greater lysis of DU145 than the tuvusertib and N-803 doublet in assays using NK cells from two of three HDs (Fig. 6A). For the NK cells from HD1, N-803 appeared to have maximized their cytolytic capacity, rendering the NK cells insensitive to further enhancement via ADCC. This observation indicates that further investigation is needed to identify biomarkers that could predict whether the triple combination will exert greater antitumor efficacy than tuvusertib and N-803 doublet and/or whether tuvusertib plus avelumab or tuvusertib plus N-803 will be the appropriate regimen.

In addition to enhancing NK cell activity, avelumab and N-803 promote T lymphocytes [26, 53, 54]. Hence, an assessment of the potential T cell modulatory effects of combining tuvusertib with these immunotherapies is warranted. Based on the increased expression of antigen processing machinery, including MHC-I (Fig. 2A,B), tuvusertib has the potential to increase the susceptibility of PCa cells to T cell targeting and ameliorate PCa’s resistance to ICB. In extension, avelumab and/or N-803 may synergize with tuvusertib to further enhance the antitumor T cell activity. While tuvusertib-induced MHC-I upregulation is contradictory to NK cell activation, MHC-I inhibitory signals can be eclipsed with sufficient ligation and signaling of activating NK receptors [9].

Tuvusertib is currently in clinical trials to assess its safety and efficacy. A first-in-human study (NCT04170153) in patients with locally advanced or metastatic unresectable solid tumors demonstrated that tuvusertib is safe and well-tolerated when given at up to 180 mg once daily. Only one unconfirmed RECIST v1.1 partial response was observed in a patient with platinum- and PARPi-resistant BRCA wild-type ovarian cancer. In this study, tuvusertib treatment did not significantly impact myeloid-derived suppressor cell (MDSC) or T and B lymphocyte levels and transiently decreased monocyte and NK cell populations at higher (≥ 130 mg) doses [5]. In another phase 1b study (NCT05396833) of the tuvusertib and avelumab combination in patients with advanced unresectable solid tumors, combination therapy achieved a RECIST v1.1 partial response in a patient with chordoma [6]. No effect on MDSC, T and B lymphocyte, monocyte, or NK cell counts was observed [6].

These early clinical findings indicate that further investigation of tuvusertib’s effect on immune cell subsets is warranted. The potential for transient decreases in NK cell populations may imply that the timing of tuvusertib and avelumab administration could be critical to the efficacy of combination therapy regimens. Such transient NK cell decreases may be countered by N-803 due to its promotion of NK and T cell activation and expansion [53, 54]. This activity and the in vivo data presented here provide a rationale for combining tuvusertib with N-803 (Fig. 7). Enzalutamide, a second generation androgen receptor antagonist used as standard of care therapy in various PCa clinical settings, has also been shown to increase NK cells and may be a viable addition to the tuvusertib + N-803 combination [55, 56].

Overall, the ongoing clinical trials suggest that tuvusertib is well tolerated and has no negative impact on immune cells. While the efficacy of tuvusertib is yet to be available, potential combination partners have to be identified for future studies. Our in vitro data demonstrate the ability of tuvusertib to sensitize PCa cells to avelumab-mediated ADCC, suggesting the combination of tuvusertib with avelumab may merit clinical investigation. Taken together, our work provides pre-clinical support for the combination of tuvusertib with ICB and/or immune-stimulating agents for PCa.

## Supplementary Information

Below is the link to the electronic supplementary material.

Supplementary file 1 (PDF 324 kb)

## Data Availability

The data that support the findings of this study are available from the corresponding author, JWH, upon reasonable request.
