# Supplementary material for: The ATR inhibitor tuvusertib (M1774) sensitizes prostate carcinoma to natural killer cell-mediated cytotoxicity, which is further augmented by the IL-15 receptor superagonist N-803
Source: Cancer Immunol Immunother. 2025 Dec 19;75(1):18. doi: 10.1007/s00262-025-04260-4 (PMC12717344; doi:10.1007/s00262-025-04260-4)
Supplement: Supplementary file 1 — (PDF 324 kb) [file 262_2025_4260_MOESM1_ESM.pdf]

Supplemental Figure S1: Tuvusertib serum level in mice receiving tuvusertib-containing chow.

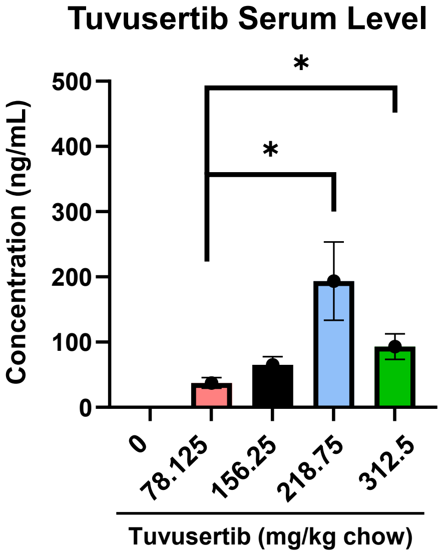

Supplemental Figure S1. Tuvusertib serum level in mice receiving tuvusertib-containing chow. On day 0, 8–12-week-old female C57BL/6 mice (Jackson Laboratory, Bar Harbor, ME, USA) were inoculated subcutaneously (s.c.) on the flank with 3.0×10^5 MC38-JAK1KO cells. Starting on day 7, tumor-bearing mice received tuvusertib-containing chow at the indicated concentrations (Research Diets, New Brunswick, NJ, USA). Serum was collected on day 14, and serum concentration of tuvusertib was determined via HPLC. Statistical tests: one-ANOVA with Tukey's post hoc test. Error bars, SEM. * $p<0.05$. ANOVA, analysis of variance; HPLC, high-performance liquid chromatography.

Supplemental Figure S2: Tuvusertib has no effect on NK cell phenotype.

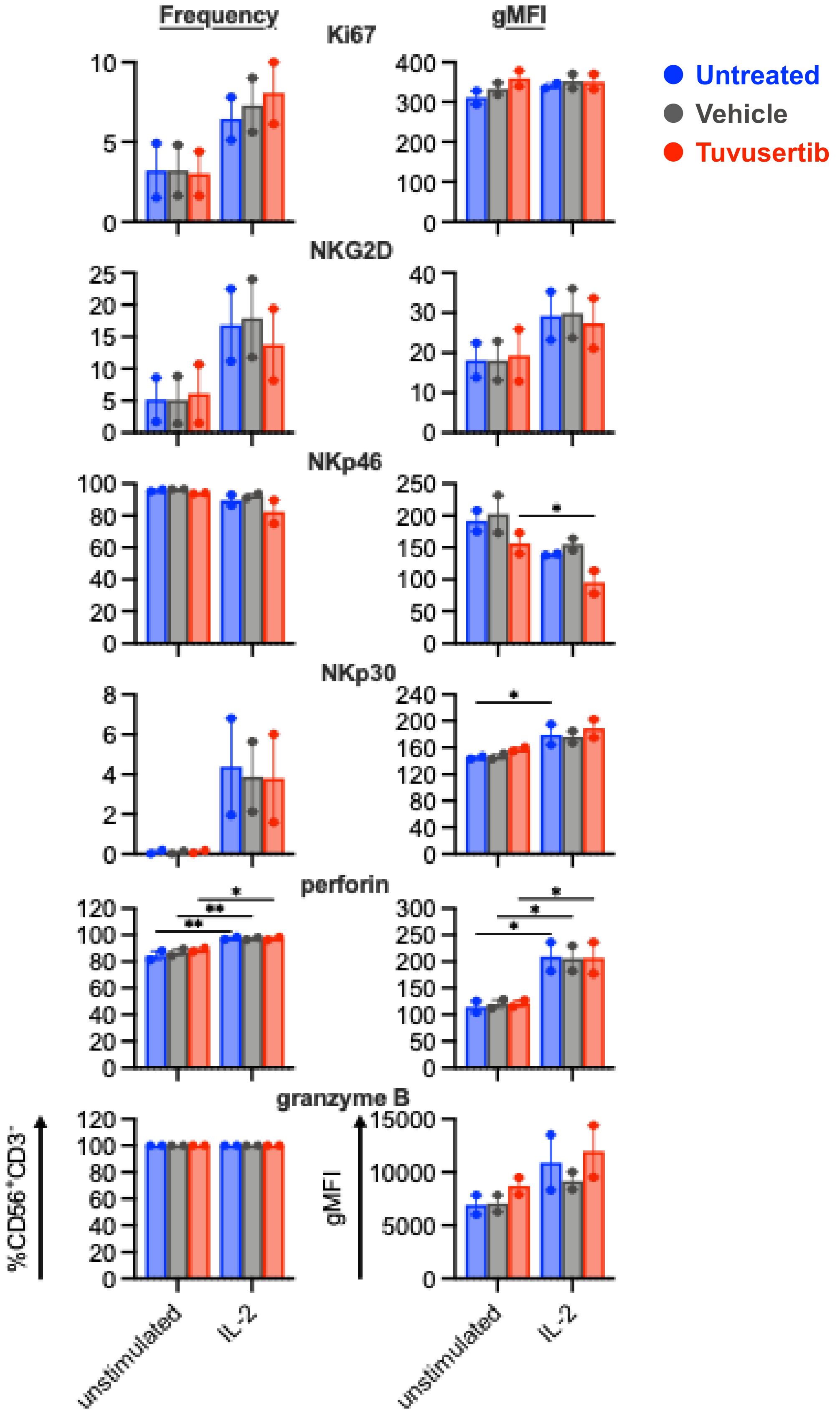

Supplemental Figure S2. Tuvusertib has no effect on NK cell phenotype. NK cells (HD8 and HD9) exposed to 0.5 μM tuvusertib and 500 IU/ml IL-2 for 48 hours were incubated with Human TruStain FcX Fc receptor blocking solution (BioLegend, San Diego, CA, USA) prior to staining with primary conjugated antibodies. The following antibodies from Biolegend were used: Ki-67-BV711 (clone Ki-67), granzyme B-FITC (GB11), and NKp46-PECy7 (9E2). The following antibodies from BD Biosciences (Franklin Lakes, NJ, USA) were used: CD3-BV605 (HIT3a), CD56-APC (B159), NKp30-BV510 (p30-15), NKG2D-BV786 (ID11), and perforin-PE (δG9). The FoxP3/transcription factor kit (eBioscience, San Diego, CA, USA) was used for intracellular staining. Live/dead fixable blue (Thermo Fisher, Waltham, MA, USA) staining was used to exclude dead cells. NK cells were identified as live/CD3−/CD56+. Data acquisition was performed using a BD LSRFortessa running FACSDiva software, and analyses were conducted using FlowJo V.10.9.0 (BD Biosciences). Percent of NK cells expressing a given marker and the gMFI of NK cells are reported. Each point represents a biological replicate. Statistical tests: two-way ANOVA with Tukey's post hoc test. Error bars, SEM. * $p<0.05$, ** $p<0.01$. ANOVA, analysis of variance. gMFI, geometric mean fluorescence intensity; HD, healthy donor; IL-2, interleukin-2; NK, natural killer; NKG2D, natural killer group 2 member D.

Supplemental Figure S3: NK-mediated lysis of tuvusertib-treated cells at several effector to target ratios.

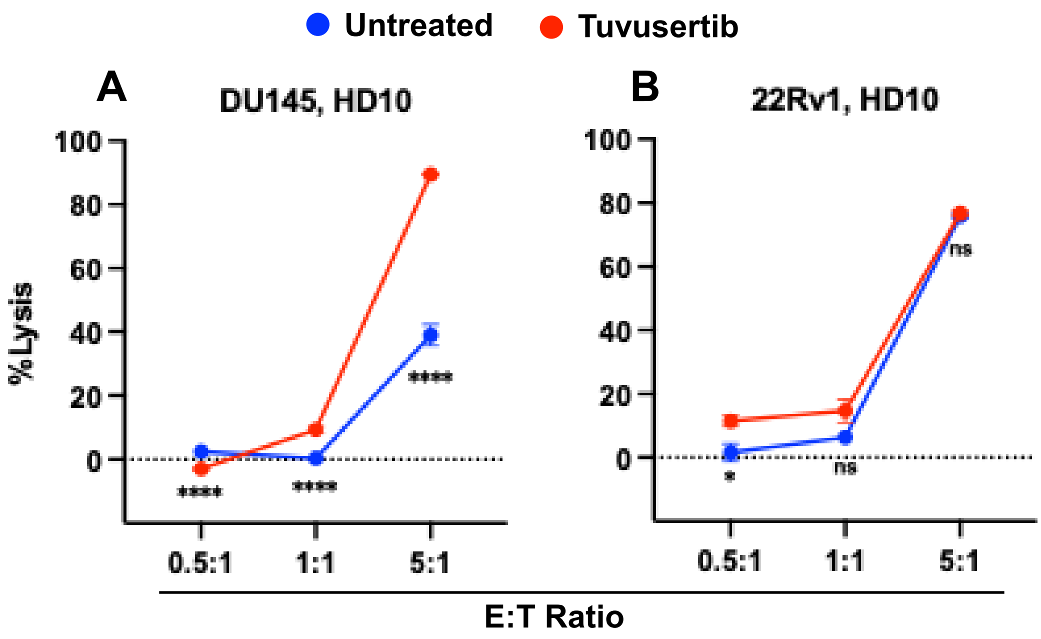

Supplemental Figure S3. NK-mediated lysis of tuvusertib-treated cells at several effector to target ratios. Prostate cancer cells (DU145 and 22Rv1) were treated with tuvusertib (0.5 µM, 48 hours), washed, and then co-incubated with NK cells at the indicated E:T ratios. Lysis of (**A**) DU145 and (**B**) 22Rv1 cells at 24 hours post-plating of effector cells. Statistical tests: Student's t-test. Error bars, SEM. ns $p \geq 0.05$, * $p<0.05$, **** $p<0.0001$. ANOVA, analysis of variance; E:T, effector to target; HD, healthy donor; NK, natural killer; ns, not significant.

Supplemental Figure S4: Tuvusertib has no detrimental effect on NK cell cytotoxic function.

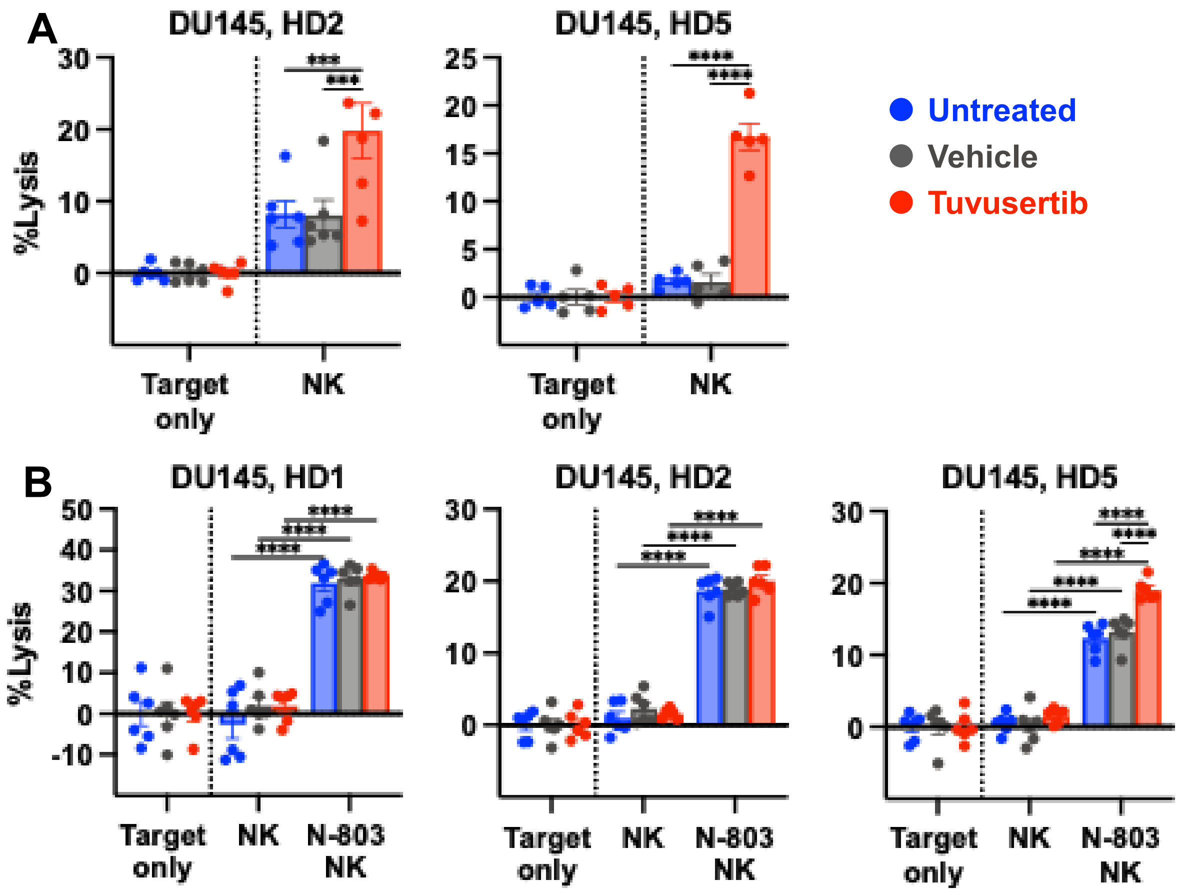

Supplemental Figure S4. Tuvusertib has no detrimental effect on NK cell cytotoxic function. To evaluate the effect of tuvusertib on the cytolytic function of NK cells, DU145 cells were co-incubated with (**A**) untreated NK cells (5:1 E:T ratio) or (**B**) N-803-treated (50 ng/ml, overnight) NK cells (0.5:1 E:T ratio) in the presence or absence of 0.5 μM tuvusertib. Lysis of DU145 cells at 24 hours post-plating of effector cells. Each point represents a technical replicate. Statistical tests: two-way ANOVA with Tukey's post hoc test. Error bars, SEM. *** p<0.001, **** p<0.0001. ANOVA, analysis of variance; E:T, effector to target; HD, healthy donor; NK, natural killer.

Supplemental Figure S5: Tuvusertib treatment did not impact TRAIL-R1 expression on DU145 and 22RV1

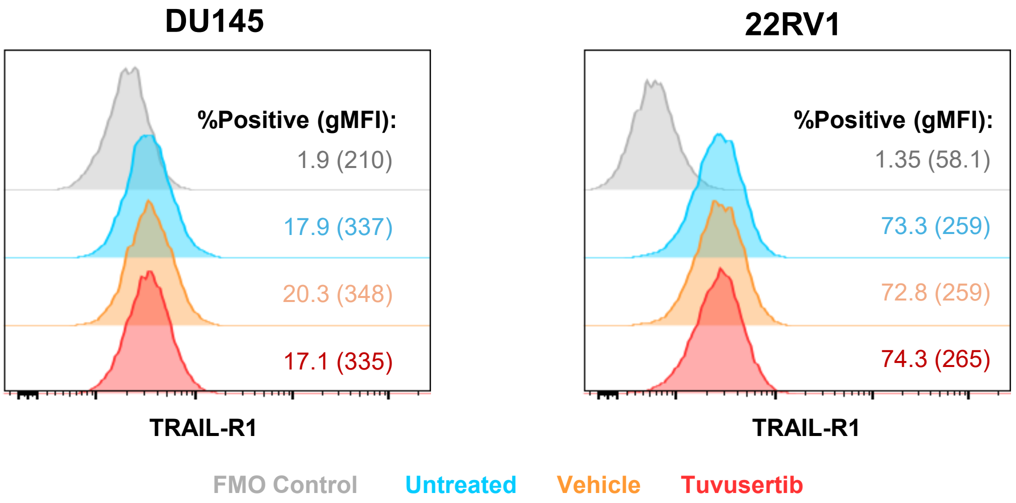

Supplemental Figure S5. Tuvusertib treatment did not impact TRAIL-R1 expression on DU145 and 22RV1. DU145 and 22Rv1 cells were treated with 0.5 µM tuvusertib for 48 hours. Afterwards, the cells were stained with far red live/dead fixable dye (Thermo Fisher, Waltham, MA, USA), exposed to Human TruStain FcX Fc receptor blocking solution (BioLegend, San Diego, CA, USA), stained with TRAIL-R1-PE (clone S35-934, BD Biosciences, Franklin Lakes, NJ, USA), and then fixed using Cytofix (BD Biosciences). Data acquisition was performed using a BD LSRFortessa running FACSDiva software, and analyses were conducted using FlowJo V.10.9.0 (BD Biosciences). Percent of live cells expressing TRAIL-R1 and gMFI of live cells are reported. FMO, fluorescence minus one; gMFI, geometric mean fluorescence intensity; TRAIL-R1, tumor-necrosis factor-related apoptosis-inducing ligand receptor 1

Supplemental Figure S6: Tuvusertib increases apoptotic cell death of DU145 cells exposed to N-803-enhanced NK cells

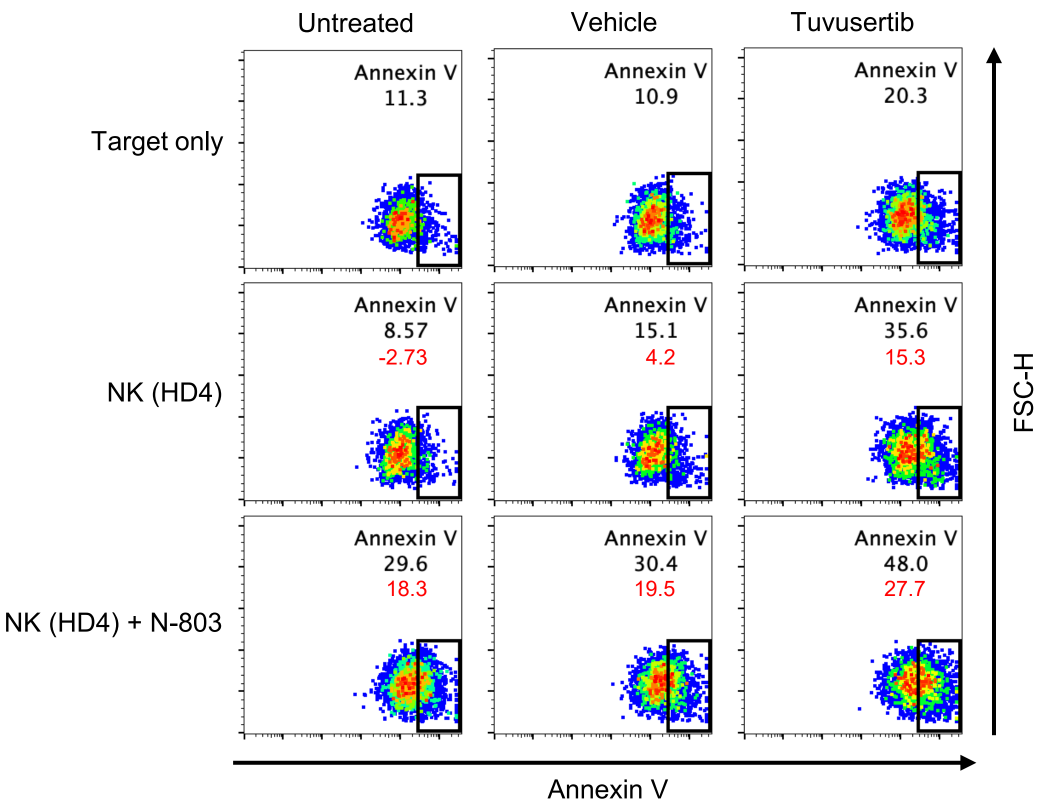

Supplemental Figure S6. Tuvusertib increases apoptotic cell death of DU145 cells exposed to N-803-enhanced NK cells. DU145 cells were treated with 0.5 µM tuvusertib for 48 hours and then stained with 1uM CellTrace Violet (Thermo Fisher, Waltham, MA, USA) per 1x10^6 cells for 20 minutes. NK cells were isolated from healthy donors using a human NK cell isolation kit (Miltenyi Biotec, Bergisch Gladbach, Germany), according to the manufacturer's protocol. Purified NK cells were rested overnight with or without 50 ng/ml N-803 prior to use. The labelled DU145 cells were co-incubated with the NK cells at 0.5:1 E:T ratio for 24 hours, after which the cells were stained with far red live/dead fixable dye (Thermo Fisher), exposed to Human TruStain FcX Fc receptor blocking solution (BioLegend, San Diego, CA, USA), stained with Annexin V-FITC (BioLegend) in the recommended buffer, and fixed using Cytofix (BD Biosciences, Franklin Lakes, NJ, USA). Data acquisition was performed using a BD LSRFortessa running FACSDiva software, and analyses were conducted using FlowJo V.10.9.0 (BD Biosciences). Cells were gated according to size, positive CellTrace Violet staining and negative live/dead fixable dye to identify intact tumor cells. Percent of these cells binding to Annexin V are reported, with the numbers in red showing the values normalized to the baseline frequency of early apoptotic cells (target only). HD, healthy donor; NK, natural killer.

Supplemental Figure S7: N-803-enhanced NK cells have increased expression of total TRAIL ligand

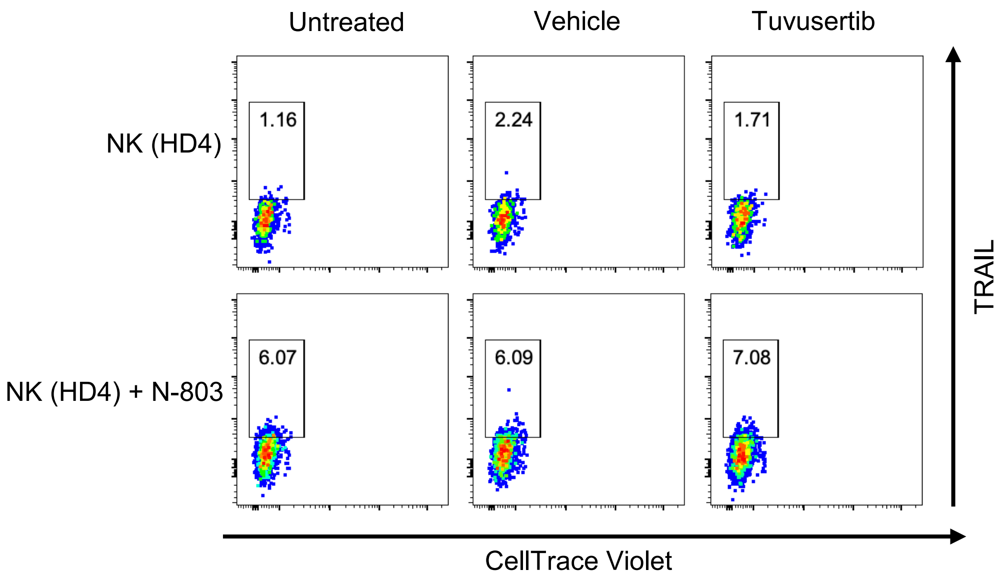

Supplemental Figure S7. N-803-enhanced NK cells have increased expression of total TRAIL ligand. DU145 cells were treated with 0.5 μM tuvusertib for 48 hours and then stained with 1 μM CellTrace Violet (Thermo Fisher, Waltham, MA, USA) per $1x10^6$ cells for 20 minutes. NK cells were isolated from healthy donors using a human NK cell isolation kit (Miltenyi Biotec, Bergisch Gladbach, Germany), according to the manufacturer's protocol. Purified NK cells were rested overnight with or without 50 ng/ml N-803 prior to use. The labelled DU145 cells were co-incubated with the NK cells at 0.5:1 E:T ratio for 4 hours in the presence of GolgiStop and GolgiPlug (BD Biosciences, Franklin Lakes, NJ, USA) using the manufacturer's recommended amounts. After which, the cells were stained with blue live/dead fixable dye (Thermo Fisher), exposed to Human TruStain FcX Fc receptor blocking solution (BioLegend, San Diego, CA, USA), permeabilized using Foxp3 Transcription Factor Staining Buffer Set (Thermo Fisher), and stained using TRAIL-APC (clone Rik-2, BioLegend). Data acquisition was performed using a BD LSRFortessa running FACSDiva software, and analyses were conducted using FlowJo V.10.9.0 (BD Biosciences). Cells were gated according to size, negative CellTrace Violet staining, and negative live/dead fixable dye to identify viable effector NK cells. Percent of these cells expressing TRAIL are reported. HD, healthy donor; NK, natural killer; TRAIL, tumor-necrosis factor-related apoptosis-inducing ligand.
